# Temporal trends and determinants of delayed care-seeking after rabies exposure: a 27,930-patient analysis in Tianjin

**DOI:** 10.3389/fpubh.2026.1911137

**Published:** 2026-08-26

**Authors:** Shuai Li, Shuangyan Liu

**Affiliations:** 1 Department of Emergency, Santan Hospital, Tianjin, China; 2 Department of Internal Medicine, Santan Hospital, Tianjin, China

**Keywords:** care-seeking delay, machine learning, post-exposure prophylaxis, rabies, XGBoost

## Abstract

**Objective:**

Prompt care-seeking after rabies exposure is essential for effective post-exposure prophylaxis (PEP). This study aimed to identify independent factors associated with care-seeking delay and to map the full PEP pathway.

**Methods:**

We retrospectively enrolled 27,930 patients attending the rabies PEP clinic at Santan Hospital, Tianjin, between February 2022 and January 2026. Delay was defined as >12 h from exposure to registration. Multivariable logistic regression, combined with XGBoost and SHAP, identified independent factors for delay and quantified their relative importance. Restricted cubic splines examined the non-linear relationship between age and delay. Subgroup analysis assessed the stability of the association of grade III exposure with more prompt care-seeking. A Sankey diagram traced the PEP pathway.

**Results:**

The delay rate was 29.7%. Eight independent factors were identified. Adults had a 34% higher risk of delay than children (aOR = 1.34, 95% CI: 1.22–1.47, *P* < 0.001). Grade III exposure was associated with more prompt care-seeking (aOR = 0.69, 95% CI: 0.65–0.73, *P* < 0.001). Previous rabies vaccination (aOR = 1.17, 95% CI: 1.11–1.23, *P* < 0.001) and inadequate wound management (aOR = 1.57, 95% CI: 1.31–1.88, *P* < 0.001) were associated with higher delay risk. Age showed a non-linear relationship with delay risk, with adults at highest risk. The Sankey diagram showed that inadequate wound management was rare in the PEP pathway, and the two-dose booster schedule achieved a much higher completion rate than the standard schedule.

**Conclusions:**

Our findings suggest that adults may face the highest risk of care-seeking delay, and that improving healthcare access for adults and promoting simplified vaccination schedules may be key to improving PEP quality.

## Introduction

Rabies is a zoonotic disease with a case fatality rate of nearly 100% and remains a major global public health threat. World Health Organization data show that most rabies deaths occur in individuals who receive post-exposure prophylaxis (PEP) too late or not at all (1). Timely and proper PEP, including wound management, vaccination, and rabies immunoglobulin (RIG) when indicated, can effectively stop disease progression. Failure to seek prompt medical attention after exposure is a key contributor to prevention failure and death (2). PEP delay rates have been reported as 60% among Dutch travelers, 10.1% among international travelers in eastern Thailand, and 27.9%−37% among animal bite victims in India (3–6).

Care-seeking delay after rabies exposure is common and leads to unnecessary health risks, anxiety, and death. Reasons are complex, including RIG availability, conflicting medical advice, age, wound characteristics, animal type, clinic hours, and inadequate education (3–6). Children are vulnerable due to curiosity and poor self-protection; they may hide injuries for fear of punishment, causing delay (7). Systematic analysis is needed to guide targeted interventions.

Most previous studies have used conventional methods, such as logistic regression and chi-square tests, which are useful for identifying factors associated with increased risk but have limited ability to capture complex interactions or nonlinear associations without strong parametric assumptions (8). Machine learning has been applied in rabies research, for instance in transmission dynamics and spatial risk mapping. However, the integration of SHAP with pathway visualization and nonlinear modeling has not been systematically used to examine care-seeking delay after rabies exposure. Consequently, the mechanisms underlying delay and the reasons for incomplete PEP remain poorly understood (9).

In summary, a comprehensive investigation of determinants of care-seeking delay and their interactions is essential for optimizing resources and implementing precise interventions. To overcome previous limitations, this study uses a large outpatient cohort. To our knowledge, this is the first study to apply machine learning algorithms to examine care-seeking behavior after rabies exposure in a human population. We also use Sankey diagrams to visualize multi-stage treatment flow and integrate clinical pathway analysis to quantify treatment behaviors. Our aim is to build a more accurate, intelligent, visual, and clinically grounded evaluation framework. This will sharpen health education, resource allocation, and policy design, thereby reducing avoidable delays and deaths, and provide high-quality local evidence to advance rabies elimination (4, 5).

## Materials and methods

This retrospective study used data from the electronic registration database of the rabies post-exposure prophylaxis PEP clinic at Santan Hospital in Nankai District, Tianjin. We screened all consecutive patients attending this clinic for animal injuries from February 2022 to January 2026.

**Inclusion criteria:**

① Exposure was classified as grade II or III according to the Technical Guidelines for Rabies Prevention and Control 2016 edition.

② The database contained complete visit records and epidemiological information for analysis.

③ All treatment time points were clearly documented.

**Exclusion criteria:**

① Exposure was grade I requiring no medical intervention.

② Vaccination records were incomplete or key data missing.

③ The attending physician determined that vaccination was unnecessary.

④ The recorded visit date preceded the injury date.

⑤ After applying these criteria, we identified 27,930 valid cases for analysis. The study protocol was approved by the Ethics Committee of Santan Hospital in Nankai District, Tianjin, approval number 20250002. Written informed consent was waived because the analysis used de-identified historical data.

### Outcome measures and endpoint definition

The primary endpoint was care-seeking delay, defined as an interval exceeding 12 h from exposure to clinic arrival and registration. The 12 h threshold used in this study was prespecified based on clinical practice and the behavioral distinction between same-day and next-day care-seeking. This cutoff is supported by several lines of evidence: the BHIVA guidelines recommend initiating treatment for high-risk bites within 12 h (10); environmental stability studies show that infectious rabies virus in saliva is no longer detectable after 12 h under summer conditions (11); viral replication kinetics indicate that viral titres peak at 12 h post-infection (12, 13); and neuroinvasion studies demonstrate that virus reaches the cerebral cortex at 12 h post-inoculation, with monoclonal antibody administered at 12 h yielding 100% survival in animal models (14, 15). However, we acknowledge that this threshold remains an operational definition rather than a universally validated standard, and sensitivity analyses using 24 h and 48 h thresholds were performed to assess the robustness of the findings. This is a binary variable, with patients presenting within 12 h serving as the reference group. The covariates extracted for analysis included age group with the reference being children under 15 years, sex, population category, previous rabies vaccination, injuring animal category, animal source, animal vaccination history, injury type, exposure site, exposure grade with the reference being grade II, inadequate wound management, and the vaccination schedule the patient subsequently received.

### Statistical analysis and modeling strategy

All data were analysed using SPSS 27.0 (IBM Corp., Armonk, NY, USA) and R 4.5.3 (R Core Team, Vienna, Austria). The dataset contained no missing values for the variables included in the analyses, as we restricted inclusion to patients with complete records for all key variables. Continuous variables following a normal distribution are presented as mean (SD) and were compared using the independent samples *t*-test; skewed variables are reported as median (*P*25, *P*75) and were compared using the Mann–Whitney *U-*test. Categorical variables are expressed as frequency (percentage) and were compared using the chi-square test. All tests were two-sided, with statistical significance set at *P* < 0.05.

Baseline characteristics were summarized for the overall cohort and compared between the delay and non-delay groups to describe the study population and the distribution of each variable. Descriptive statistics are as described above, with group comparisons made using the appropriate tests.

To identify independent factors associated with care-seeking delay, we performed univariate and multivariable logistic regression. Each candidate variable was first entered into a univariate model; those with *P* < 0.1 were then included in the multivariable model using a stepwise selection procedure. The outcome was care-seeking delay (binary: >12 h vs. ≤ 12 h), and results are reported as odds ratios (OR) with 95% confidence intervals (CI).

To retain the continuous nature of the care-seeking interval and to complement the primary binary analysis, we additionally fitted an accelerated failure time (AFT) model with care-seeking time in hours as the continuous outcome, as a sensitivity analysis. The same variable selection strategy was applied (univariate *P* < 0.1 for entry into the multivariable model). Results are expressed as acceleration factors (AF), where AF > 1 indicates prolonged care-seeking time and AF < 1 indicates shortened care-seeking time.

To quantify the relative contribution of each variable to care-seeking delay, we applied the XGBoost algorithm combined with the SHAP method, using the same set of predictors as in the logistic regression. Categorical variables were converted to numeric values using label encoding (as.numeric(as.factor()) in R), with each categorical variable entered as a single feature. SHAP values were calculated using the shapviz package, and variable importance was assessed by the mean absolute SHAP value. The XGBoost model was fitted with fixed hyperparameters (nrounds = 200, eta = 0.03, max_depth = 4, subsample = 0.8, colsample_bytree = 0.8) and a random seed of 123, trained on the full dataset without train-test split or cross-validation, as the primary purpose was variable importance ranking rather than developing a predictive tool. Importance bar plots, beeswarm plots, and dependence plots were generated to visualize variable importance and the direction of effects.

To explore non-linear relationships between continuous variables and care-seeking delay, we fitted restricted cubic splines, using care-seeking delay as the outcome and continuous variables (e.g., age) as the main exposure, with adjustment for covariates. Dose-response curves were plotted to test for non-linear trends.

To assess whether the effect of the core exposure variable (exposure grade) was consistent across subgroups, we conducted stratified analyses by age group, sex, population category, prior vaccination history, injuring animal category, animal source, animal vaccination history, injury type, exposure site, and inadequate wound management. Forest plots were used to display effect estimates for each subgroup, and interaction *P* values were calculated to test for effect modification. Interactions were tested by including product terms between exposure grade and each stratification variable in the logistic regression model. Finally, to visualize the complete patient flow from exposure to vaccination completion and to identify critical nodes in the PEP pathway, we constructed age-stratified Sankey diagrams, mapping patient transitions across five sequential nodes: exposure grade, wound management, care-seeking delay, vaccination schedule, and full vaccination. These diagrams allowed visual comparison of flow distributions and pathway patterns across age groups.

## Results

### Study population inclusion and exclusion process

**Figure 1:**
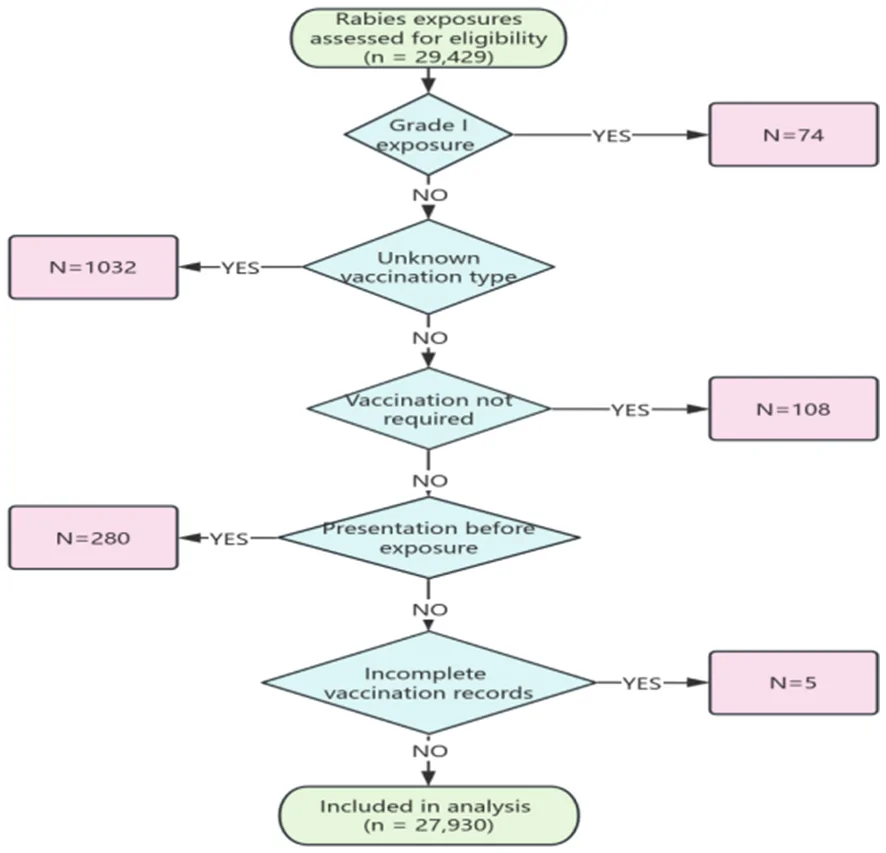
Research flowchart.

Of 29,429 patients initially assessed, 27,930 were included after sequential application of predefined criteria. The screening flow is shown in Figure 1. Exclusions comprised: grade I exposure (*n* = 74), where intact skin contact carries negligible transmission risk and PEP is not indicated; unknown vaccination type (*n* = 1,032). Among these patients, some may have received their first dose at another facility and attended our clinic only for subsequent doses, but the available system data did not allow us to reliably identify the time of the first visit for any patient in this category. As the primary outcome was the interval from exposure to the first clinic visit, these patients were excluded to avoid misclassifying a subsequent dose as the initial visit; judged not to require vaccination (*n* = 108), including cases of incorrect grade I classification, exposure to non-WHO-listed high-risk animals, or psychological fear without actual injury; visit date recorded before exposure (*n* = 280), representing pre-exposure prophylaxis for travel or occupational risk rather than PEP; and incomplete vaccination records (*n* = 5), precluding valid analysis.

### Baseline characteristics and factors associated with care-seeking delay

Among the 27,930 patients enrolled in this study, 8,301 patients (29.7%) were assigned to the delay group and 19,629 patients (70.3%) to the non-delay group. The two groups showed significant differences in age distribution, population category, previous rabies vaccination, injuring animal category, animal vaccination history, injury type, exposure grade, inadequate wound management, and complete vaccination (Table 1).

**Table 1:** Baseline characteristics of the 27,930 patients who received rabies post-exposure prophylaxis.

Variables	Total (*n* = 27,930)	0 (*n* = 19,629)	1 (*n* = 8,301)	Statistic	*P*
Age at exposure	33.45 ± 18.01	33.76 ± 18.33	32.70 ± 17.20	*t* = 4.62	**<0.001**
Weight	62.49 ± 18.78	62.39 ± 18.95	62.70 ± 18.36	*t* = −1.27	0.203
Treatment delay	22.41 ± 638.28	2.93 ± 2.92	68.48 ± 1,169.56	*t* = −5.11	**< 0.001**
Age, *n*(%)				χ^2^ = 73.13	**< 0.001**
<15 years	4,839 (17.33)	3,533 (18.00)	1,306 (15.73)		
15–60 years	2,0242 (72.47)	13,945 (71.04)	6,297 (75.86)		
≥60 years	2,849 (10.20)	2,151 (10.96)	698 (8.41)		
Gender, *n*(%)				χ^2^ = 1.64	0.200
Female	16,306 (58.38)	11,508 (58.63)	4,798 (57.80)		
Male	11,624 (41.62)	8,121 (41.37)	3,503 (42.20)		
Population category, *n*(%)				χ^2^ = 8.27	**0.041**
General population	20,393 (73.01)	14,284 (72.77)	6,109 (73.59)		
High-risk occupations	324 (1.16)	226 (1.15)	98 (1.18)		
Students	7,104 (25.44)	5,030 (25.63)	2,074 (24.98)		
Very high-risk occupations	109 (0.39)	89 (0.45)	20 (0.24)		
Previous rabies vaccination, *n*(%)				χ^2^ = 24.61	**< 0.001**
No	18,489 (66.20)	13,173 (67.11)	5,316 (64.04)		
Unknown	82 (0.29)	57 (0.29)	25 (0.30)		
Yes	9,359 (33.51)	6,399 (32.60)	2,960 (35.66)		
Injuring animal category, *n*(%)				χ^2^ = 73.38	**< 0.001**
Bats	44 (0.16)	34 (0.17)	10 (0.12)		
Cats/Dogs	26,412 (94.56)	18,414 (93.81)	7,998 (96.35)		
Other animals	1,474 (5.28)	1,181 (6.02)	293 (3.53)		
Animal source, *n*(%)				χ^2^ = 5.62	0.131
Domestic	22,356 (80.04)	15,686 (79.91)	6,670 (80.35)		
Stray	4,766 (17.06)	3,350 (17.07)	1,416 (17.06)		
Wild	205 (0.73)	143 (0.73)	62 (0.75)		
Animal vaccination history, *n*(%)				χ^2^ = 34.86	**< 0.001**
No	5,947 (21.29)	4,018 (20.47)	1,929 (23.24)		
Unknown	14,804 (53.00)	10,422 (53.09)	4,382 (52.79)		
Yes	7,179 (25.70)	5,189 (26.44)	1,990 (23.97)		
Injury type, *n*(%)				χ^2^ = 20.39	**< 0.001**
Bite/Scratch/Lick on broken skin	27,230 (97.49)	19,168 (97.65)	8,062 (97.12)		
Contact with intact skin	14 (0.05)	8 (0.04)	6 (0.07)		
Open wound/mucosa contact	319 (1.14)	189 (0.96)	130 (1.57)		
Other	367 (1.31)	264 (1.34)	103 (1.24)		
Exposure site, *n*(%)				χ^2^ = 1.78	0.182
Head/Neck	1,199 (4.29)	822 (4.19)	377 (4.54)		
Trunk/Limbs	26,731 (95.71)	18,807 (95.81)	7,924 (95.46)		
Exposure grade, *n*(%)				χ^2^ = 177.27	**< 0.001**
Grade II	14,996 (53.69)	10,032 (51.11)	4,964 (59.80)		
Grade III	12,934 (46.31)	9,597 (48.89)	3,337 (40.20)		
Inadequate wound management, *n*(%)				χ^2^ = 35.35	**< 0.001**
Yes	780 (2.79)	623 (3.17)	157 (1.89)		
No	27,150 (97.21)	19,006 (96.83)	8,144 (98.11)		
Complete vaccination, *n*(%)				χ^2^ = 5.26	**0.022**
No	16,260 (58.22)	11,341 (57.78)	4,919 (59.26)		
Yes	11,670 (41.78)	8,288 (42.22)	3,382 (40.74)		

Bold values indicate statistical significance.

Multivariable logistic regression identified several independent factors associated with care-seeking delay (Table 2). Adults had a 34% higher risk of delay compared with children (OR = 1.34, 95% CI: 1.22–1.47). Prior rabies vaccination was associated with a 17% increased delay risk (OR = 1.17, 95% CI: 1.11–1.23), and open wound or mucosal contact with a 91% increase (OR = 1.91, 95% CI: 1.52–2.41). Inadequate wound management was also associated with higher delay risk (OR = 1.57, 95% CI: 1.31–1.88). Factors associated with lower delay risk included very high-risk occupations (OR = 0.54, 95% CI: 0.33–0.88), stray animal source (OR = 0.90, 95% CI: 0.84–0.97), unknown animal source (OR = 0.73, 95% CI: 0.60–0.88), unknown animal vaccination history (OR = 0.93, 95% CI: 0.87–0.99), vaccinated animal status (OR = 0.79, 95% CI: 0.73–0.86), and grade III exposure (OR = 0.69, 95% CI: 0.65–0.73).

**Table 2:** Univariate and multivariable logistic regression analyses of factors associated with delayed healthcare seeking after rabies exposure.

Variables	Univariate					Multivariate				
	β	S.E	Z	*P*	OR (95% CI)	β	S.E	Z	*P*	OR (95% CI)
Age										
<15 years					1.00 (Reference)					1.00 (Reference)
15–60 years	0.20	0.04	5.60	**<0.001**	1.22 (1.14–1.31)	0.29	0.05	6.09	**< 0.001**	1.34 (1.22–1.47)
≥60 years	−0.13	0.05	−2.40	**0.016**	0.88 (0.79–0.98)	−0.01	0.07	−0.15	0.878	0.99 (0.87–1.13)
Gender										
Female							1.00 (Reference)			
Male	0.03	0.03	1.28	0.200	1.03 (0.98–1.09)					
Population category										
General population					1.00 (Reference)					1.00 (Reference)
High-risk occupations	0.01	0.12	0.11	0.910	1.01 (0.80–1.29)	0.24	0.13	1.81	0.070	1.27 (0.98–1.64)
Students	−0.04	0.03	−1.21	0.227	0.96 (0.91–1.02)	0.06	0.04	1.60	0.109	1.07 (0.99–1.15)
Very high-risk occupations	−0.64	0.25	−2.60	**0.009**	0.53 (0.32–0.85)	−0.62	0.25	−2.49	**0.013**	0.54 (0.33–0.88)
Previous rabies vaccination										
No					1.00 (Reference)					1.00 (Reference)
Unknown	0.08	0.24	0.35	0.729	1.09 (0.68–1.74)	0.11	0.24	0.44	0.661	1.11 (0.69–1.79)
Yes	0.14	0.03	4.96	**<0.001**	1.15 (1.09–1.21)	0.16	0.03	5.53	**< 0.001**	1.17 (1.11–1.23)
Injuring animal category										
Bats					1.00 (Reference)					
Cats/Dogs	0.39	0.36	1.08	0.279	1.48 (0.73–2.99)					
Other animals	−0.17	0.37	−0.47	0.642	0.84 (0.41–1.73)					
Animal source										
Domestic					1.00 (Reference)					1.00 (Reference)
Stray	−0.01	0.03	−0.17	0.864	0.99 (0.93–1.06)	−0.10	0.04	−2.84	**0.004**	0.90 (0.84–0.97)
Unknown	−0.22	0.09	−2.36	**0.018**	0.80 (0.66–0.96)	−0.32	0.10	−3.27	**0.001**	0.73 (0.60–0.88)
Wild	0.02	0.15	0.13	0.899	1.02 (0.76–1.38)	0.02	0.15	0.12	0.907	1.02 (0.75–1.38)
Animal vaccination history										
No					1.00 (Reference)					1.00 (Reference)
Unknown	−0.13	0.03	−4.01	**<0.001**	0.88 (0.82–0.93)	−0.07	0.03	−2.17	**0.030**	0.93 (0.87–0.99)
Yes	−0.22	0.04	−5.87	**< 0.001**	0.80 (0.74–0.86)	−0.23	0.04	−5.80	**< 0.001**	0.79 (0.73–0.86)
Injury type										
Bite/Scratch/Lick on broken skin					1.00 (Reference)					1.00 (Reference)
Contact with intact skin	0.58	0.54	1.07	0.284	1.78 (0.62–5.14)	0.65	0.55	1.19	0.232	1.92 (0.66–5.59)
Open wound/mucosa contact	0.49	0.11	4.29	**<0.001**	1.64 (1.31–2.05)	0.65	0.12	5.57	**< 0.001**	1.91 (1.52–2.41)
Other	−0.08	0.12	−0.64	0.520	0.93 (0.74–1.17)	−0.01	0.12	−0.11	0.914	0.99 (0.78–1.24)
Exposure site										
Head/Neck					1.00 (Reference)					
Trunk/Limbs	−0.08	0.06	−1.33	0.182	0.92 (0.81–1.04)					
Exposure grade										
Grade II					1.00 (Reference)					1.00 (Reference)
Grade III	−0.35	0.03	−13.29	**<0.001**	0.70 (0.67–0.74)	−0.37	0.03	−13.67	**< 0.001**	0.69 (0.65–0.73)
Inadequate wound management										
No					1.00 (Reference)					1.00 (Reference)
Yes	0.53	0.09	5.88	**<0.001**	1.70 (1.42–2.03)	0.45	0.09	4.93	**< 0.001**	1.57 (1.31–1.88)
Weight	0.00	0.00	1.26	0.208	1.00 (1.00–1.00)					

Bold values indicate statistical significance.

### AFT model analysis using continuous care-seeking time

To further utilize the continuous nature of the care-seeking interval, we constructed an AFT model with care-seeking time (in hours) as the continuous outcome variable, as a sensitivity analysis to complement the primary findings. Variables with *P* < 0.1 in univariate analysis were entered into the multivariable model. Results are presented as AF; AF > 1 indicates prolonged care-seeking time, while AF < 1 indicates shortened care-seeking time (Table 3).

**Table 3:** Univariate and multivariable AFT model analysis of care-seeking time after rabies exposure.

Variable	Level	Univariate_ Overall_ *P*	Univariate_AF_CI	Multivariate_ Overall_ *P*	Multivariate_ AF_ CI
Exposure_grade	Grade II (Ref)	<0.001	1.00	<0.001	1.00
	Grade III		0.728 (0.703–0.753)		0.725 (0.700–0.750)
Age_group	Child (Ref)	<0.001	1.00	<0.001	1.00
	Adult		1.134 (1.083–1.187)		1.142 (1.091–1.196)
	older adults		0.935 (0.874–1.001)		0.966 (0.902–1.034)
Gender	Female (Ref)	0.428	1.00		
	Male		1.014 (0.979–1.050)		
Population_category	General population (Ref)	0.768	1.00		
	Students		0.994 (0.955–1.034)		
	High-risk occupations		1.062 (0.903–1.248)		
	Very high-risk occupations		0.773 (0.586–1.020)		
Previous_rabies_vaccination	Vaccinated (Ref)	<0.001	1.00	<0.001	1.00
	Unvaccinated		0.890 (0.858–0.924)		0.879 (0.848–0.912)
	Unknown		1.116 (0.810–1.536)		1.104 (0.804–1.514)
Injuring_animal_category	Other animals (Ref)	<0.001	1.00	<0.001	1.00
	Cat/Dog		1.423 (1.318–1.538)		1.422 (1.316–1.537)
	Bat		1.313 (0.845–2.040)		1.289 (0.831–1.999)
Animal_source	Domestic (Ref)	0.736	1.00		
	Stray		1.008 (0.962–1.055)		
	Unknown		0.918 (0.815–1.034)		
	Wild		1.012 (0.826–1.241)		
Animal_vaccination_history	Vaccinated (Ref)	<0.001	1.00	<0.001	1.00
	Unknown		1.146 (1.099–1.194)		1.203 (1.154–1.255)
	Unvaccinated		1.250 (1.188–1.314)		1.262 (1.200–1.328)
Injury_type	Intact skin contact (Ref)	<0.001	1.00	<0.001	1.00
	Open wound/mucosa contact		1.252 (0.570–2.752)		1.319 (0.602–2.887)
	Other		0.795 (0.362–1.743)		0.761 (0.348–1.662)
	Bite/scratch/lick on broken skin		0.830 (0.384–1.794)		0.733 (0.340–1.578)
Exposure_site	Trunk/Limbs (Ref)	0.795	1.00		
	Head/Neck		0.880 (0.337–2.302)		
	Other sites		1.041 (0.956–1.134)		
Inadequate wound management	Standard (Ref)	<0.001	1.00	<0.001	1.00
	Non-standard		1.410 (1.270–1.566)		1.322 (1.190–1.468)

AFT, accelerated failure time model; AF, acceleration factor; CI, confidence interval. Multivariable model included only variables with *P* < 0.1 in univariate analysis. Reference group AF = 1.00. AF < 1 indicates shorter care-seeking time; AF>1 indicates longer care-seeking time. Model fit: *R*^2^ = 0.006, RMSE = 28.36 h, MAE = 10.03 h.

In the multivariable AFT model, patients with grade III exposure had a care-seeking time 0.725 times that of grade II patients (95% CI: 0.700–0.750, *P* < 0.001), corresponding to a 27.5% reduction. Adults had a care-seeking time 1.142 times that of children (95% CI: 1.091–1.196, *P* < 0.001), representing a 14.2% prolongation, whereas older adults did not differ significantly from children (AF = 0.966, 95% CI: 0.902–1.034, *P* = 0.134). Patients with no prior vaccination had a care-seeking time 0.879 times that of vaccinated patients (95% CI: 0.848–0.912, *P* < 0.001). Dog or cat injuries were associated with a 1.422-fold prolongation compared with other animal injuries (95% CI: 1.316–1.537, *P* < 0.001). Relative to patients injured by vaccinated animals, those with unknown or unvaccinated animal status had AFs of 1.203 (95% CI: 1.154–1.255, *P* < 0.001) and 1.262 (95% CI: 1.200–1.328, *P* < 0.001), respectively. Patients with non-standard wound management had a care-seeking time 1.322 times that of those with standard management (95% CI: 1.190–1.468, *P* < 0.001). These findings were consistent with the primary logistic regression results, supporting the robustness of the observed associations.

### Sensitivity analysis using alternative delay thresholds

To assess whether the choice of the 12-h cutoff influenced our findings, we repeated the multivariable logistic regression using 24 h and 48 h thresholds as alternative definitions of delay (full results in Supplementary Table S1). Core variables, including exposure grade, age group (15–60 years), inadequate wound management, and animal source, showed consistent directions of association across all three thresholds, supporting the robustness of the primary conclusions. However, some variables showed direction changes at the 24 h threshold, and sparse data prevented estimation for certain subgroups at the 48 h threshold. Overall, the primary analysis using the 12 h threshold remained stable across alternative definitions.

### Machine learning feature importance assessment

Multivariable logistic regression can identify independent factors but does not readily permit direct comparison of their relative importance. To address this, we applied the XGBoost algorithm combined with the SHAP method to quantify each variable's contribution to care-seeking delay. The analysis used the same set of predictors as the logistic regression, with the binary delay outcome, and was intended primarily for variable importance ranking rather than for building a clinical prediction tool.

**Figure 2:**
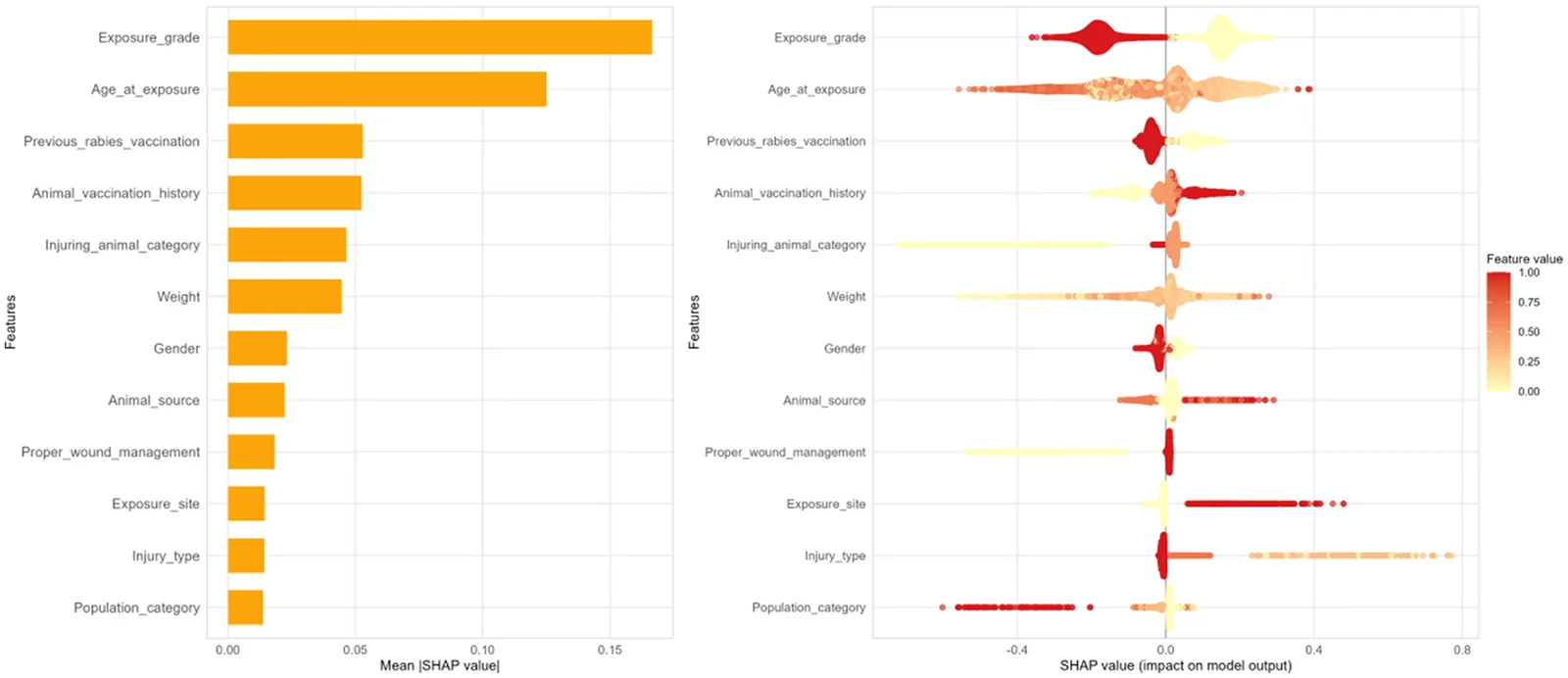
SHAP feature importance and individual impact distribution for factors associated with treatment delay. **(Left)** Feature importance bar plot. The length of each bar represents the mean absolute SHAP value for the corresponding feature; longer bars indicate greater influence on care-seeking delay. Features are arranged in descending order of importance. **(Right)** Beeswarm plot of feature impact distribution. Each dot represents an individual patient. The horizontal position indicates the direction of contribution to delay risk: positive values (right side) increase the risk of delay, while negative values (left side) decrease it. Color represents the original value of the feature, with red indicating higher values and yellow indicating lower values.

The SHAP importance bar plot (Figure 2, left) showed that exposure grade contributed most, followed by age at exposure, previous vaccination history, and animal vaccination history. This ranking was consistent with findings from both logistic regression and the AFT model.

**Figure 3:**
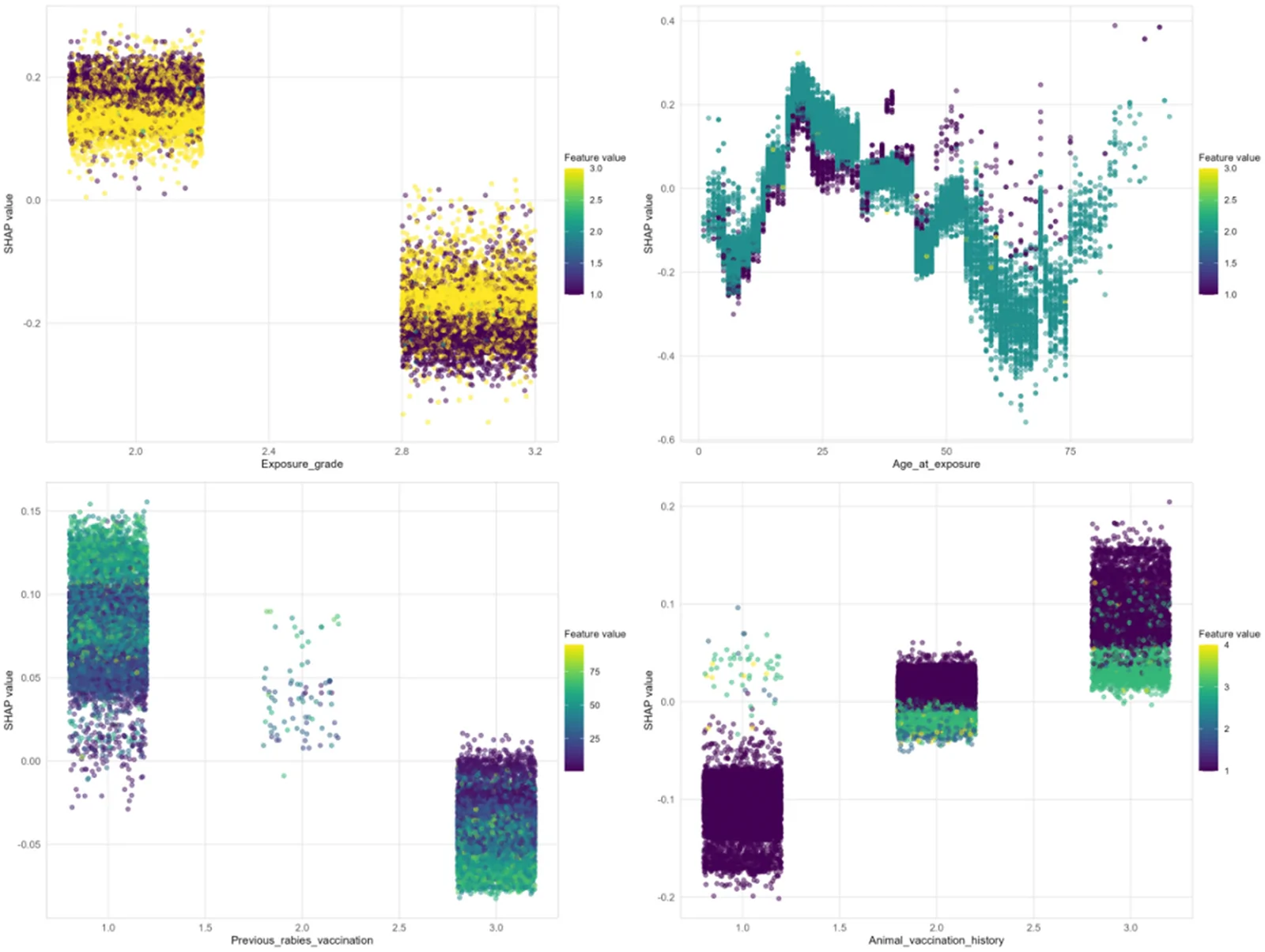
Non-linear dependence relationships between key features and the risk of treatment delay. The figure shows the dependence of SHAP values on four key features: age at exposure, previous rabies vaccination, animal vaccination history, and exposure grade. In each panel, the horizontal axis represents the feature value and the vertical axis represents the corresponding SHAP value. SHAP values greater than zero indicate an increased risk of delay, while values less than zero indicate a decreased risk. Dot color represents the value of another feature that interacts with the feature shown. The fitted curve reveals potential non-linear association patterns.

In the beeswarm plot (Figure 2, right) and the dependence plot (Figure 3), higher exposure grade values were associated with negative SHAP values, and SHAP values declined with increasing exposure grade, indicating that grade III exposure was linked to lower delay risk. For age at exposure, higher values were predominantly associated with positive SHAP values. The dependence plot showed an arch shaped curve: SHAP values rose with age in younger groups, peaked in adults, and then declined, suggesting a nonlinear relationship between age and delay risk. Higher values of previous vaccination history were associated with positive SHAP values, and the dependence plot showed that vaccinated individuals had higher SHAP values, indicating increased delay risk. For animal vaccination history, lower values were associated with positive SHAP values, and the dependence plot showed higher SHAP values among those injured by unvaccinated animals, suggesting greater delay risk when the animal was not vaccinated. These findings were consistent with the logistic regression results. Exposure grade and age at exposure were consistently identified as the core features shaping care-seeking delay, and the non-linear pattern of age warranted further exploration.

At the optimal threshold of 0.25, the XGBoost model achieved a sensitivity of 0.875, specificity of 0.254, accuracy of 0.439, and F1 score of 0.481. These metrics are reported for transparency only, as the model was used for exploratory variable ranking rather than predictive purposes. The model was therefore used primarily as a supplementary tool for variable importance ranking.

### Association between exposure grade and care-seeking delay

**Figure 4:**
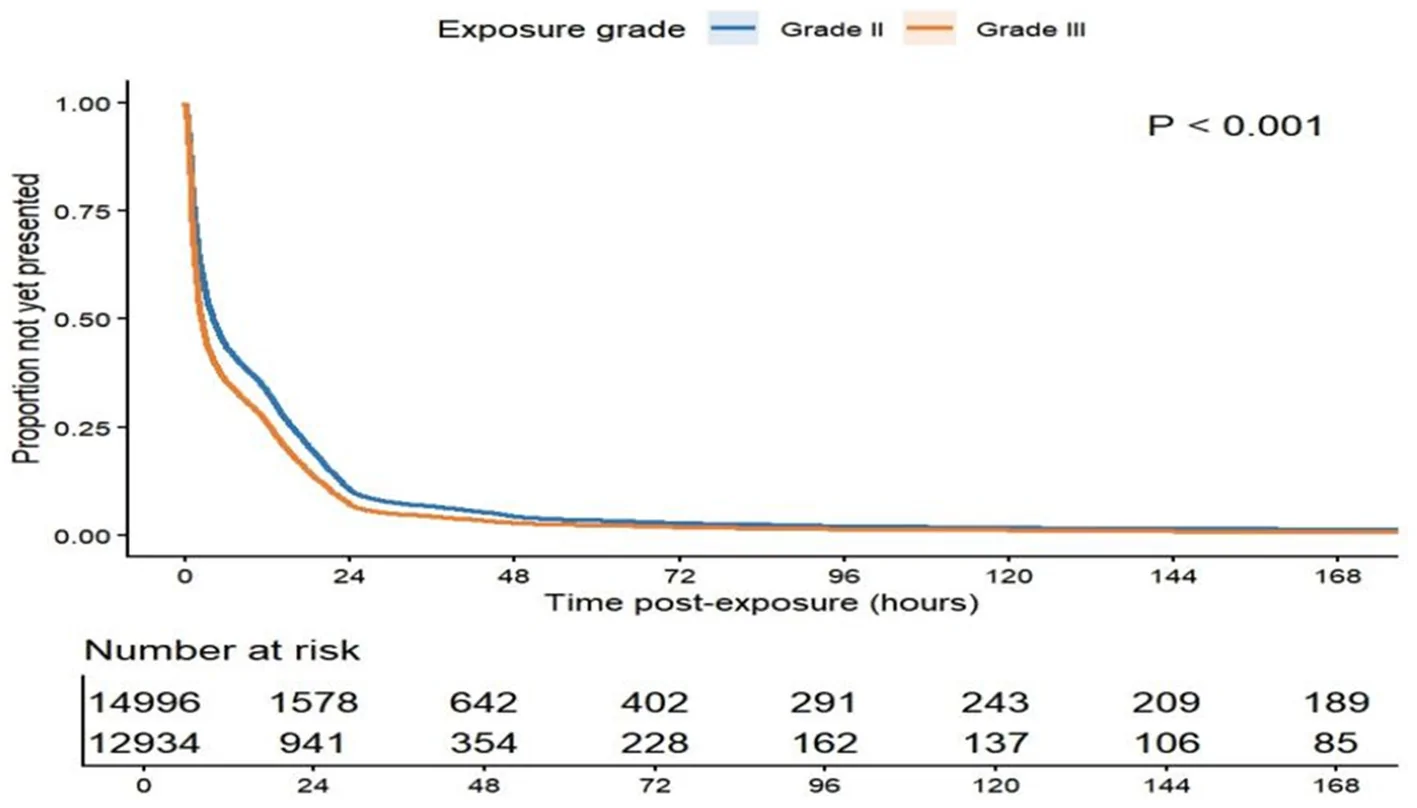
Time-dependent proportion of patients not seeking care after rabies exposure, stratified by exposure grade.

SHAP importance analysis ranked exposure grade first, and logistic regression confirmed its association with more prompt care-seeking. We illustrated the temporal difference in care-seeking behavior between the two exposure grades using cumulative distribution curves (Figure 4). The curve for grade III exposure lay consistently below that for grade II exposure at all time points, indicating that the proportion of patients who had not yet sought care was consistently lower in the grade III group. The difference between the two curves was statistically significant (χ^2^ = 1,710.53, *P* < 0.001). These findings indicate that patients with grade III exposure sought care more quickly than those with grade II exposure, likely reflecting greater perceived severity and clinical urgency.

### Independent association between age and care-seeking delay

Trend analysis used children as the reference group. In unadjusted Model 1, adults had a higher delay risk (OR = 1.22, 95% CI 1.14–1.31) and older adults a lower risk (OR = 0.88, 95% CI 0.79–0.98). In Model 2, after adjusting for sex and weight, the OR for adults was 1.34 (95% CI 1.22–1.47), while the OR for older adults was not statistically significant. In Model 3, with further adjustment for population category, previous rabies vaccination, injuring animal category, animal vaccination history, exposure grade, and inadequate wound management, the OR for adults was 1.43 (95% CI 1.28–1.59), and older adults did not differ significantly from children (Table 4).

**Table 4:** Trend analysis and multivariable adjustment for the association between age group and treatment delay.

Variables	Model 1		Model 2		Model 3	
	OR (95% CI)	*P*	OR (95% CI)	*P*	OR (95% CI)	*P*
Age group						
<15 years	1.00 (Reference)		1.00 (Reference)		1.00 (Reference)	
15–60 years	1.22 (1.14–1.31)	**<0.001**	1.34 (1.22–1.47)	**< 0.001**	1.43 (1.28–1.59)	**< 0.001**
≥60 years	0.88 (0.79–0.98)	**0.016**	0.96 (0.85–1.08)	0.488	1.05 (0.91–1.20)	0.505

Model 1: Unadjusted model.

Model 2: Adjusted for sex and body weight.

Model 3: Adjusted for above + patient category, prior rabies vaccination history, category of injuring animal, animal immunization status, exposure grade, and inadequate wound management.

Bold values indicate statistical significance.

**Figure 5:**
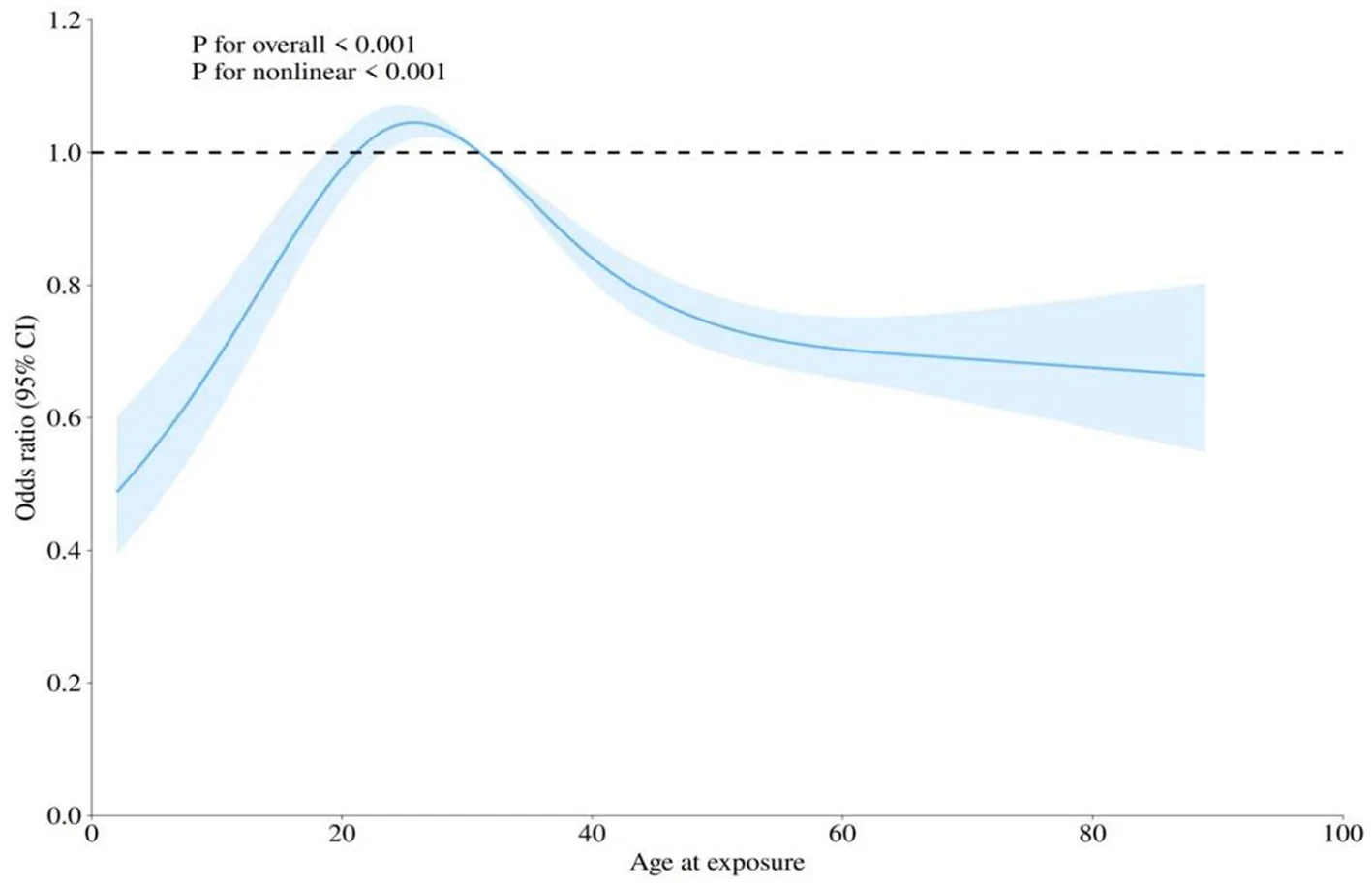
Restricted cubic spline analysis of the association between age at exposure and the risk of treatment delay.

Restricted cubic spline analysis (Figure 5), adjusted for sex, population category, previous rabies vaccination, injuring animal category, animal vaccination history, exposure grade, and inadequate wound management, showed a non-linear relationship between age and delay risk (overall *P* < 0.001, non-linearity *P* < 0.001). With age 31 years as reference, risk rose from childhood, peaked at 40–50 years, and declined thereafter.

### Subgroup analysis

**Figure 6:**
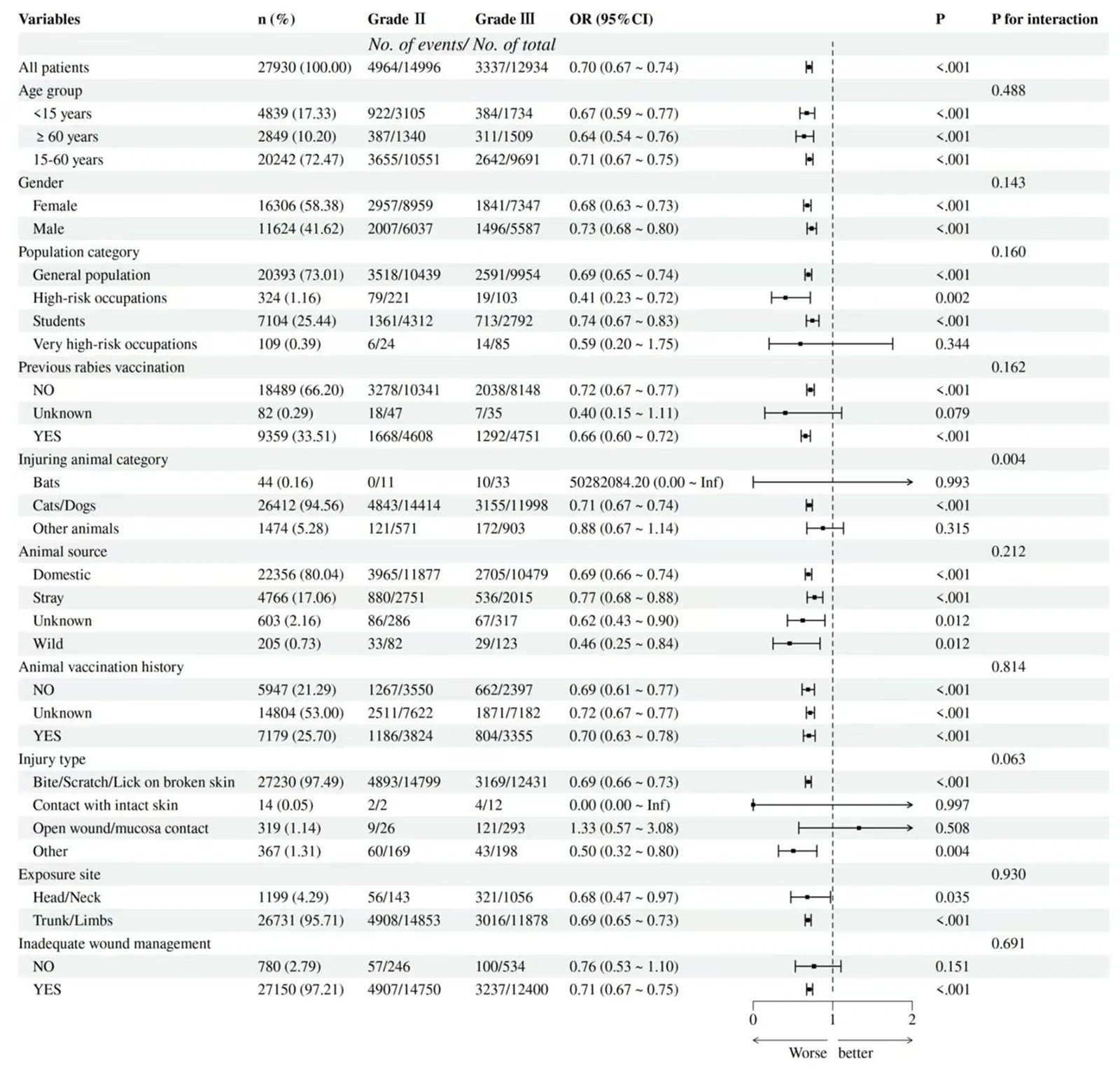
Subgroup analysis of key factors associated with delayed healthcare seeking after rabies exposure.

Stratified analyses by age, sex, population category, previous rabies vaccination, injuring animal category, animal source, animal vaccination history, injury type, exposure site, and inadequate wound management were performed (Figure 6). Interaction *P* values were adjusted for multiple comparisons using the Bonferroni method, with a corrected significance threshold of 0.005 (0.05–10). After correction, only the interaction between grade III exposure and injuring animal category remained statistically significant (*P* = 0.004). In the dog/cat injury subgroup, the OR was 0.71 (95% CI 0.67–0.74); in the other animal injury subgroup, the OR was 0.88 (95% CI 0.67–1.14). For all other stratification variables, adjusted interaction *P* values exceeded 0.005. In all subgroups, the OR for grade III exposure was below 1.

### Pathway analysis of key PEP nodes

**Figure 7:**
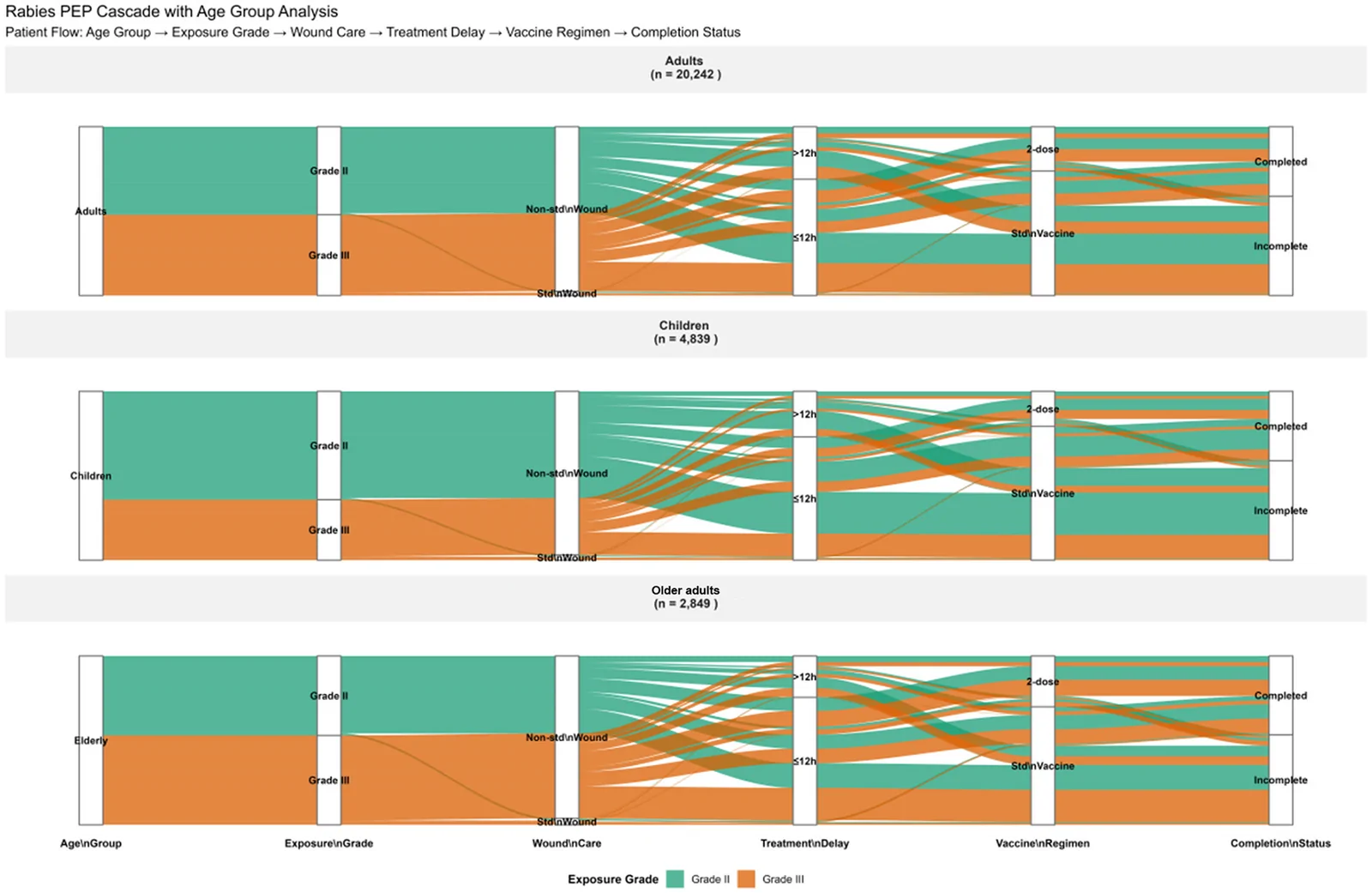
Age-stratified Sankey diagram of patient pathways through the rabies post-exposure prophylaxis cascade.

Age-stratified Sankey diagrams mapped patient flow across five nodes: exposure grade, wound management, care-seeking delay, vaccination schedule, and complete vaccination (Figure 7). At the wound management node, most flows converged toward proper management, with a very narrow inadequate management stream. At the care-seeking delay node, the delay flow was broader for adults than for children and older adults. Patients with grade III exposure showed a larger proportion of non-delay flow than those with grade II. At the vaccination schedule node, patients with complete prior vaccination entered the two-dose booster schedule; others entered the standard schedule. At the complete vaccination node, nearly all flow from the two-dose booster schedule reached completion, whereas a substantial portion of the standard schedule flow did not.

**Figure 8:**
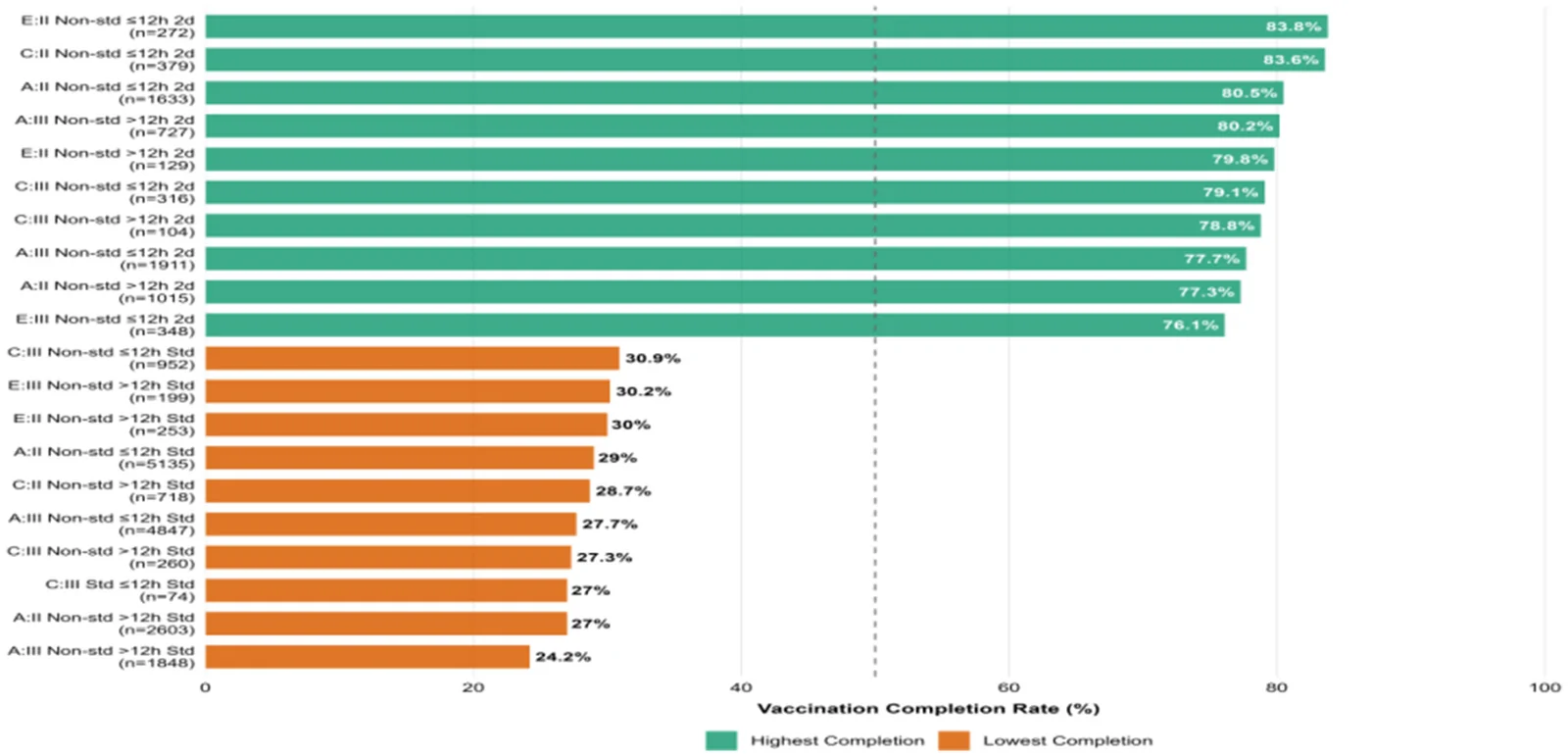
Comparison of the ten patient pathways with the highest and lowest completion rates of rabies post-exposure prophylaxis.

We compared the 10 highest and 10 lowest completion pathways (Figure 8, Table 5). All high-completion pathways used the two-dose booster schedule, with rates from 76% to 84%. All low-completion pathways used the standard schedule, with rates from 24% to 31%. Children and older adults were more common in high-completion pathways, and most of these patients did not delay care. Adults dominated low-completion pathways, and the proportion of delayed care was substantial.

**Table 5:** Characteristics of patient pathways with the highest and lowest completion rates of rabies post-exposure prophylaxis.

Pathway category	Rank	Patient pathway	Number of patients	Completion rate (%)
Protective	1	O:II Non-std ≤ 12h 2d	272	83.8
Protective	2	C:II Non-std ≤ 12h 2d	379	83.6
Protective	3	A:II Non-std ≤ 12h 2d	1,633	80.5
Protective	4	A:III Non-std >12h 2d	727	80.2
Protective	5	O:II Non-std >12h 2d	129	79.8
Protective	6	C:III Non-std ≤ 12h 2d	316	79.1
Protective	7	C:III Non-std >12h 2d	104	78.8
Protective	8	A:III Non-std ≤ 12h 2d	1,911	77.7
Protective	9	A:II Non-std >12h 2d	1,015	77.3
Protective	10	O:III Non-std ≤ 12h 2d	348	76.1
High-risk	1	A:III Non-std >12h Std	1,848	24.2
High-risk	2	A:II Non-std >12h Std	2,603	27
High-risk	3	C:III Std ≤ 12h Std	74	27
High-risk	4	C:III Non-std >12h Std	260	27.3
High-risk	5	A:III Non-std ≤ 12h Std	4,847	27.7
High-risk	6	C:II Non-std >12h Std	718	28.7
High-risk	7	A:II Non-std ≤ 12h Std	5,135	29
High-risk	8	O:II Non-std >12h Std	253	30
High-risk	9	O:III Non-std >12h Std	199	30.2
High-risk	10	C:III Non-std ≤ 12h Std	952	30.9

C, Children; A, Adults; O:Older adults; II/III, WHO exposure grade; Non-std, Non-standard wound management; Std, Standard wound management; 12h/>12h, Treatment delay; 2d, 2-dose booster regimen; Std, Standard vaccination regimen (5-dose Essen or 4-dose Zagreb).

## Discussion

This large retrospective cohort study of 27,930 rabies-exposed individuals yielded three main findings. First, the rate of care-seeking delay (≥12 h after injury) was 29.7%, and eight independent factors were identified: age, population category, previous rabies vaccination, injuring animal category, animal vaccination history, injury type, exposure grade, and inadequate wound management. Second, age showed a strong non-linear association with delay risk, with adults facing the highest risk. Third, PEP pathway analysis revealed critical bottlenecks: inadequate wound management was extremely rare, and the completion rate of the standard schedule (five-dose Essen or four-dose Zagreb) was far below that of the two-dose booster schedule. Full PEP completion diverged markedly by age and by timeliness of care. These findings highlight modifiable obstacles to timely and complete PEP in urban China and suggest potential targets for public health interventions, though the clinical importance of individual factors varies according to the strength of their associations.

The observed delay rate of 29.7% aligns with reports from low- and middle-income countries where 20–40% of patients present beyond 24 h (16–18). Even in an urban setting with relatively good medical access, nearly one in three exposed individuals failed to initiate PEP within the 12 h threshold used in this study. Prompt wound care and early PEP are recognized as effective in reducing the risk of central nervous system invasion by rabies virus; previous studies have linked each hour of delay to increased infection risk (3, 19, 20). Beyond Asia, similar patterns have been documented in other endemic regions. In Uganda, a study of 376 dog bite victims found that nearly 40% delayed seeking PEP, with low education level (no formal education: adjusted PR = 4.06) and low socioeconomic status identified as key risk factors (21). In Rwanda, a retrospective cross-sectional study of 412 dog bite victims in Nyagatare District reported that 39% delayed PEP; rural residence (aOR = 3.54) and lack of health insurance (aOR = 4.40) were the main barriers (22). In Thailand, a 7 year retrospective study of 528 international travelers at an emergency center in the east found that 10.1% delayed PEP; factors associated with delay included age 35–60 years (aOR = 3.08), superficial wounds (aOR = 2.86), and a single wound (aOR = 1.88) (4); another study in Bangkok reported that over 40% of travelers delayed the first vaccine dose by more than 24 h (23). These cross-regional comparisons suggest that while determinants share common features, their relative importance varies by setting, underscoring the need for context-specific interventions.

Sensitivity analyses using 24 h and 48 h thresholds showed that core variables, including exposure grade, age, wound management, and animal source, were directionally consistent across thresholds, supporting the robustness of the primary findings. However, some variables showed direction changes at different cutoffs. For example, prior rabies vaccination was associated with higher delay risk at the 12 h threshold (OR = 1.17) but became associated with lower delay risk at the 24 h threshold (OR = 0.84). One possible explanation is behavioral heterogeneity among previously vaccinated individuals. Some may delay care because they believe they are protected, contributing to the risk at 12 h; others, who have basic rabies knowledge, may still present within 24 h, contributing to the protective effect at the later threshold. Similarly, animal vaccination status reversed direction: unvaccinated animal status was protective at 12 h (OR = 0.79), likely because patients perceived higher risk from unvaccinated animals and sought care promptly. However, at 24 h this association reversed direction, becoming associated with higher risk (OR = 1.58), possibly because those who did not attend within 12 h may have lowered their vigilance if the animal appeared healthy or the wound seemed minor. These interpretations remain speculative and require further investigation.

Some of the statistically significant associations identified in this study had modest effect sizes. for example, prior rabies vaccination (OR = 1.17) and unknown animal source (OR = 0.73). While these findings reached statistical significance due to the large sample size, their clinical implications should be interpreted with caution. The most clinically relevant associations were observed for age (OR = 1.34), grade III exposure (OR = 0.69), and inadequate wound management (OR = 1.57), which showed effect sizes of greater magnitude and were consistent across multiple analytical approaches.

A consistent finding across our analyses was the elevated delay risk among adults aged 31–50 years. Earlier research often singled out children and rural populations, but our findings suggest that urban working adults may be more likely to delay care-seeking (24–26). The inverted U-shaped relationship between age and delay, confirmed by restricted cubic spline analysis, challenges the linear assumption of earlier studies. This pattern was further reflected in age-related disparities in PEP completion: children and older adults were more likely to complete the full course, whereas adults clustered in low-compliance pathways. Adults in this age group may face competing work demands, costs associated with time off, or a tendency to underestimate minor injuries. These interpretations are speculative, as these factors were not directly measured in our study. The combination of adult status, delayed care, and assignment to a traditional schedule is associated with a high-risk profile for PEP interruption.

Grade III exposures involve high-risk scenarios such as head or face injuries, penetrating bites, mucous membrane contamination, or bat contact. Patients may perceive these injuries as more severe, which could be associated with earlier attendance (27). However, the age composition of the grade III group, which contained a higher proportion of adults, may partly explain the attenuation of this tendency, given that adults themselves tend to be more prone to delay. This pattern is consistent with the idea that perceived severity influences health-seeking behavior, although this was not directly measured in our study. The age composition of the grade III group—which contained a higher proportion of adults—highlights the need to consider demographic characteristics when interpreting exposure-related differences in care-seeking.

Children usually present with guardians, facilitating timely care. Older adults, despite mobility limitations, have more discretionary time and higher health awareness. Age was identified as a core confounder in the exposure grade-delay relationship. Failure to adjust for age could overestimate the independent effect of exposure grade on care-seeking.

Our study showed that inadequate wound management was uncommon across all age groups and exposure grades; the majority of patients followed the standard protocol of washing with soapy water for 15 min followed by disinfection. However, when inadequate management did occur, it was associated with increased delay risk (OR = 1.57). This association is clinically relevant, as proper wound management reduces viral load and is a cornerstone of rabies prevention (27). The low prevalence of inadequate care suggests effective clinical practices at our center, yet the strong link between inadequate management and delay warrants continued attention to this minority subgroup, particularly given the potential for adverse outcomes when wound care is suboptimal. Possible contributing factors for the minority of patients with inadequate wound management may include lack of immediate access to appropriate wound-care facilities before presentation, or insufficient awareness of the importance of wound irrigation among the small proportion of non-adherent individuals (4, 28). These findings point to the potential value of systematic remediation across multiple domains: public education, healthcare worker training, and provision of wound care materials.

The two-dose booster schedule was associated with a markedly higher completion rate than the traditional five-dose Essen or four-dose Zagreb regimens. This aligns with evidence supporting simplified schedules (29, 30). Fewer clinic visits enhance adherence, and the WHO position paper recommends simplified schedules especially in resource-limited settings (31, 32). Completion of traditional multi-dose schedules ranged between 24 and 31%. Repeated return visits are time-intensive and burdensome, and working adults are particularly prone to attrition due to work, transport, and forgetfulness. Patients who delay seeking care are even more likely to forgo completion (4, 29, 33). The two-dose schedule dramatically reduces follow-up visits, fits urban adults' daily routines, and holds promise for broader implementation.

Sankey pathway analysis also revealed age-related disparities in PEP completion. Children and older adults were more likely to complete the full course, whereas adults clustered in low-compliance pathways, mirroring the age distribution of delay. The combination of adult status, delayed care, and assignment to a traditional schedule is associated with a high-risk profile for PEP interruption. Interventions to support adherence in this group, such as reminder systems or reduced-visit regimens, have been suggested in previous studies and may warrant further evaluation (34–36).

These findings have actionable implications. Targeted health education and outreach efforts may be particularly relevant for working adults (4, 37–40). Systematic efforts to improve wound management practices across healthcare facilities, including enhanced public awareness of immediate wound care, standardized training for healthcare personnel, and adequate supplies for wound irrigation, may be warranted (4, 40). Broader use of simplified vaccination schedules, supported by policy and training for healthcare workers, could improve adherence. Complementary strategies such as reminder systems and extended clinic hours have been suggested in previous studies to facilitate completion among working adults, although these were not evaluated in our study. An age-stratified approach, with attention to adults who have grade III high-risk exposures, may also be beneficial (38–41).

Strengths of this study include the large sample size of 27,930 cases, broad baseline data, and advanced analytical methods including restricted cubic splines, and Sankey diagrams. The study also incorporated two complementary outcomes: care-seeking delay and full PEP compliance, covering the entire continuum from exposure to complete prevention.

We acknowledge several limitations. First, this was a single-center study in urban Tianjin, where PEP access is good; our findings may not apply to rural or underserved settings where transport, clinic hours, or vaccine supply may pose additional barriers. Second, the 12 h cutoff for delay was prespecified but remains an operational rather than universally validated threshold. We performed sensitivity analyses using 24 h and 48 h cutoffs, which supported the robustness of the primary findings for core variables. However, we cannot exclude the possibility that different thresholds might yield different results for some secondary variables. Third, we lacked data on socioeconomic factors such as education, income, insurance, and travel distance, which may have introduced residual confounding. Fourth, the retrospective design precludes causal inference. Fifth, several exposure-related variables, including the timing of exposure, previous vaccination history, and animal vaccination status, relied on patient-reported information and may be subject to recall bias. While exposure timing was recorded at consultation and cross-checked against registration timestamps, which may have reduced inaccuracy for patients presenting promptly, recall error remains a possibility, particularly for those with longer delays. This is an inherent limitation of registry-based studies. Sixth, although we used XGBoost for variable importance ranking, the model was not externally validated using an independent dataset, which limits the generalisability of the machine learning findings. Future multi-center urban-rural studies are needed to validate our findings, alongside qualitative and implementation research to explore barriers and test interventions.

## Conclusion

Our study suggests that adults may represent the population with the highest delay risk for delayed PEP seeking after rabies exposure in this cohort. It indicates that the association of grade III exposure with timely care may differ by age, and that simplified schedules were associated with higher completion in this cohort. Through targeted health education, improved wound management, wider use of simplified schedules, and age-stratified strategies, post-exposure rabies risk in urban areas could potentially be reduced and the comprehensive rabies control framework strengthened. The strongest evidence from this study supports interventions targeting adults, wound management practices, and the use of simplified vaccination schedules. Our findings support the global goal of zero human deaths from dog-mediated rabies.

## Data Availability

The raw data supporting the conclusions of this article will be made available by the authors, without undue reservation.

## References

[1] Rupprecht CE Abela-Ridder B Abila R Amparo AC Banyard A Blanton J . Towards rabies elimination in the Asia-Pacific region: from theory to practice. Biologicals. (2020) 64:83–95. doi: 10.1016/j.biologicals.2020.01.008 32089431

[2] Thumbi SM Blumberg L le Roux K Salahuddin N Abela B. A call to accelerate an end to human rabies deaths. Lancet. (2022) 400:2261–4. doi: 10.1016/S0140-6736(22)02487-4 36528379 PMC9754655

[3] de Feij M Luppino FS Visser LG Vlot JA. Delays in rabies post-exposure prophylaxis abroad. J Travel Med. (2025) 32:taaf111. doi: 10.1093/jtm/taaf111 41123543

[4] Singkam N Sapsirisavat V Chanduan J Piyabenjarad P Limpitigranon P Wisitthipakdeekul S . Delayed and incomplete rabies post-exposure prophylaxis among international travelers: a seven-year retrospective study at an emergency center in eastern Thailand. Travel Med Infect Dis. (2025) 67:102873. doi: 10.1016/j.tmaid.2025.102873 40602738

[5] Gupta RK Dhoundiyal A Choudhary S Chandra R Shakya L. Factors associated with delay in initiating postexposure prophylaxis for rabies prevention in a district of Madhya Pradesh, India: 2023. J Family Med Prim Care. (2025) 14:4563–7. doi: 10.4103/jfmpc.jfmpc_302_25 41403543 PMC12704991

[6] Khan IA Bashar MDA Mohsin S Shrivastava DK. Prevalence of delayed initiation of rabies postexposure prophylaxis and factors influencing it among animal bite victims from Eastern Uttar Pradesh, India. Trans R Soc Trop Med Hyg. (2024) 118:399–404. doi: 10.1093/trstmh/trae002 38324406

[7] Rabies Prevention and Control Committee of the Chinese Preventive Medicine Association; Branch of Animal Injury Treatment; China Association for Disaster and Emergency Rescue Medicine. Expert consensus on rabies post-exposure prophylaxis in children (2025 edition). Zhonghua Liu Xing Bing Xue Za Zhi. (2026) 47:381–. Chinese. 10.3760/cma.j.cn112338-20260108-00025 41876168

[8] Shen T Welburn SC Sun L Yang GJ. Progress towards dog-mediated rabies elimination in PR China: a scoping review. Infect Dis Poverty. (2023) 12:30. doi: 10.1186/s40249-023-01082-3 37024944 PMC10077633

[9] Verdoes L Luppino FS Wallinga PJ Visser PLG. Delayed rabies post-exposure prophylaxis treatment among Dutch travellers during their stay abroad: a comprehensive analysis. J Travel Med. (2021) 28:taaa240. doi: 10.1093/jtm/taaa240 33403393

[10] Geretti AM Brook G Cameron C . British HIV association guidelines on the use of vaccines in HIV-positive adults 2015. HIV Med. (2016) 17:s2–81. doi: 10.1111/hiv.12424 27568789

[11] Zhang M Zhang N Xu X . Stability analysis of rabies virus in the environment. J Virol Methods. (2026) 346:115437. doi: 10.1016/j.jviromet.2026.115437 42462824

[12] Wang Y He H Li J . Rabies virus-induced autophagy is dependent on viral load in BV2 cells. Front Microbiol. (2021) 12:595678. doi: 10.3389/fmicb.2021.595678 34113320 PMC8186530

[13] Sarkar S Biligiri KK Vats N . Participation of host cell proteins in inclusion bodies of non-segmented RNA virus infected cells: a molecular insight. Virol J. (2025) 22:282. doi: 10.1186/s12985-025-02784-w 40826091 PMC12362845

[14] Dietzschold B Kao M Zheng YM . Delineation of putative mechanisms involved in antibody-mediated clearance of rabies virus from the central nervous system. Proc Natl Acad Sci USA. (1992) 89:7252–6. doi: 10.1073/pnas.89.15.7252 1496020 PMC49684

[15] Shankar V Dietzschold B Koprowski H. Direct entry of rabies virus into the central nervous system without prior local replication. J Virol. (1991) 65:2736–8. doi: 10.1128/jvi.65.5.2736-2738.1991 2016778 PMC240640

[16] Adhikari A Adhikari S Adhikari A Magar SR Khanal S Bastola S . A retrospective analysis of rabies post-exposure prophylaxis in Kapilvastu District, Nepal. BMC Public Health. (2026) 26:1474. doi: 10.1186/s12889-026-27015-x 41826898 PMC13147678

[17] Khazaei S Shirzadi MR Amiri B Pourmozafari J Ayubi E. Epidemiologic aspects of animal bite, rabies, and predictors of delay in post-exposure prophylaxis: a national registry-based study in Iran. J Res Health Sci. (2023) 23:e00583. doi: 10.34172/jrhs.2023.118 37571954 PMC10422135

[18] Kumar I Parmar D Panesar S Dhamnetiya D. Epidemiological profile and factors associated with the delay in initiation of post exposure prophylaxis of animal bite cases at the tertiary care hospital, New Delhi: a hospital based study. Virus Dis. (2025) 36:377–83. doi: 10.1007/s13337-025-00928-8 PMC12635000 41281981

[19] Rabies Prevention and Control Committee of the Chinese Preventive Medicine Association; Animal Injury Treatment Branch of China Association for Disaster and Emergency Rescue Medicine. Expert consensus on the clinical application of anti-rabies virus monoclonal antibody (2025 edition). Zhonghua Liu Xing Bing Xue Za Zhi. (2025) 46:1721–30. doi: 10.3760/cma.j.cn112338-20250808-00568 41443678

[20] Whitehouse ER Mandra A Bonwitt J Beasley EA Taliano J Rao AK. Human rabies despite post-exposure prophylaxis: a systematic review of fatal breakthrough infections after zoonotic exposures. Lancet Infect Dis. (2023) 23:e167–74. doi: 10.1016/S1473-3099(22)00641-7 36535276 PMC11403119

[21] Kisaka S Makumbi F Majalija S . Delays in initiating rabies post-exposure prophylaxis among dog bite victims in Wakiso and Kampala districts, Uganda. AAS Open Res. (2022) 4:49. doi: 10.12688/aasopenres.13311.3 36419540 PMC9648361

[22] Mugwaneza D Ndagijimana A Hakizayezu F . Factors associated with delayed rabies post-exposure prophylaxis among dog bite victims in Nyagatare District, Rwanda, 2017 to 2019. Rwanda J Med Health Sci. (2023) 6:61–70. doi: 10.4314/rjmhs.v6i1.8 40567929 PMC12110464

[23] Charoenwisedsil R Soravipukuntorn T. Panyatanakun K . Travel-related potential rabid animal post-exposure consultation at the Thai Travel Clinic, Hospital for Tropical Diseases, Bangkok, Thailand. Travel Med Infect Dis. (2025) 66:102870. doi: 10.1016/j.tmaid.2025.102870 40516802

[24] Regassa BT Tosisa W Eshetu D Mulu A Hundie GB. Incidence, risk factors, and control of Rabies in Ethiopia: a systematic review and meta-analysis. PLoS Negl Trop Dis. (2025) 19:e0012874. doi: 10.1371/journal.pntd.0012874 40106430 PMC11922250

[25] Yin WW Wang CL Chen QL Dong GM Li YH Zhu WY . Expert consensus on rabies exposure prophylaxis. Zhonghua Yu Fang Yi Xue Za Zhi. (2019) 53:668–79. doi: 10.3760/cma.j.issn.0253-9624.2019.07.004 31288336

[26] Bates JE Chen MH Constine LS. Radiation-associated coronary disease in young cancer survivors: the beat goes on; we must preserve it. JACC CardioOncol. (2021) 3:393–6. doi: 10.1016/j.jaccao.2021.08.002 34604799 PMC8463725

[27] Liu S Liu C Chen QJ Zhu ZG Lyu XJ Wang CL . Interpretation of the national regulation for the rabies exposure prophylaxis (2023 edition). Zhonghua Liu Xing Bing Xue Za Zhi. (2023) 44:1497–506. doi: 10.3760/cma.j.cn112338-20230905-00127 37875436

[28] Lodha L Gupta S Ashwini MA Chandel S Mani RS. Rabies despite post-exposure prophylaxis in high-burden settings: vaccine failure or gaps in healthcare delivery? Clin Infect Dis. (2025) 6:ciaf662. doi: 10.1093/cid/ciaf662 41350240

[29] Auerswald H Maestri A Touch S In S Ya N Heng B . Side-by-side comparative study of the immunogenicity of the intramuscular and intradermal rabies post-exposure prophylaxis regimens in a cohort of suspected rabies virus exposed individuals. Clin Infect Dis. (2023) 77:910–6. doi: 10.1093/cid/ciad304 37337899 PMC10506778

[30] Jin F Zhu L Wang Y Qin G Tian Y Xie Y . Corrigendum to “Randomized, blind, controlled phase III clinical trial: assessing the immunogenicity and safety of freeze-dried human rabies vaccine (vero cell) with a 4-dose regimen (2-1-1) in a 10-60 year-old demographic” [Vaccine (2024) 26059]. Vaccine. (2024) 42:126128. doi: 10.1016/j.vaccine.2024.07.029 39019659

[31] World Health Organization. Rabies vaccines: WHO position paper, April 2018—Recommendations. Vaccine. (2018) 36:5500–3. doi: 10.1016/j.vaccine.2018.06.061 30107991

[32] Nadal D Bote K Masthi R Narayana A Ross Y Wallace R . Rabies post-exposure prophylaxis delivery to ensure treatment efficacy and increase compliance. IJID One Health. (2023) 1:100006. doi: 10.1016/j.ijidoh.2023.100006 38152594 PMC10752235

[33] Al-Hinai A Al Yazidi L Al Jamei A Shalaby A Al-Said K Al Rawahi Y . Cytomegalovirus colitis in 2 patients with kindler syndrome. Pediatr Infect Dis J. (2025) 44:e270. doi: 10.1097/INF.0000000000004765 39961129

[34] Pustilnik HN Cerqueira GA Fontes JHM Meira DA Medrado-Nunes GS da Cunha BLB . Barbed versus conventional suture for spinal surgery: a systematic review and meta-analysis. World Neurosurg. (2025) 193:241–55. doi: 10.1016/j.wneu.2024.10.117 39515462

[35] Milkman KL Patel MS Gandhi L Graci HN Gromet DM Ho H . A megastudy of text-based nudges encouraging patients to get vaccinated at an upcoming doctor's appointment. Proc Natl Acad Sci U S A. (2021) 118:e2101165118. doi: 10.1073/pnas.2527439122 33926993 PMC8157982

[36] Lu R Lin J Zhou Y Chen Q Fan Z Wu S . Rabies vaccination adherence and associated factors among rabies-exposed patients in Shenzhen, China: a hospital-based cross-sectional study. Epidemiol Infect. (2024) 152:e15. doi: 10.1017/S0950268824000049 38195536 PMC10894897

[37] Kallas-Silva L Couto MT Soares MEM Ferreira-Silva SN Avelino-Silva VI. Myths and misinformation associated with vaccine incompleteness: a survey study. Patient Educ Couns. (2025) 131:108556. doi: 10.1016/j.pec.2024.108556 39579518

[38] Yamabhai J Cusripituck P Mingbualuang T Sangkachai N Sakchainanon W Tungwongjulaniam C . A persona-based exploration of rabies post-exposure prophylaxis seeking behavior and its implication for communication strategic planning: evidence from Thailand. One Health. (2025) 20:100980. doi: 10.1016/j.onehlt.2025.100980 39931353 PMC11808511

[39] Madhumathi J Sinha R Veeraraghavan B Walia K. Use of “Social Media”-an option for spreading awareness in infection prevention. Curr Treat Options Infect Dis. (2021) 13:14–31. doi: 10.1007/s40506-020-00244-3 33519303 PMC7826144

[40] Sonpar A Hundal CO Totté JEE Wang J Klein SD Twyman A . Multimodal strategies for the implementation of infection prevention and control interventions-update of a systematic review for the WHO guidelines on core components of infection prevention and control programmes at the facility level. Clin Microbiol Infect. (2025) 31:948–57. doi: 10.1016/j.cmi.2025.01.011 39863071

[41] Alpar S Colak FU Kaya B Yilmaz S. The impact of media on surge capacity in emergency departments: a study on rabies vaccination uptake. BMC Emerg Med. (2025) 25:102. doi: 10.1186/s12873-025-01270-4 40597662 PMC12210710

