# Supplementary material for: Temporal trends and determinants of delayed care-seeking after rabies exposure: a 27,930-patient analysis in Tianjin
Source: Front Public Health. 2026 Aug 26;14:1911137. doi: 10.3389/fpubh.2026.1911137 (PMC13522977; doi:10.3389/fpubh.2026.1911137)
Supplement: Supplementary file 1 [file Table_1.docx]

Supplementary Table S1. Sensitivity analysis of factors associated with care‑seeking delay using 24‑hour and 48‑hour thresholds.

| **Variable** | **Subgroup** | **12.hour_aOR** | **24.hour_aOR** | **48.hour_aOR** |
| --- | --- | --- | --- | --- |
| Age group | <15 years | 1.00 (Reference) | 1.00 (Reference) | 1.00 (Reference) |
|  | 15–60 years | 1.34 (1.22 – 1.47) | 1.15 (1.02 – 1.29) | 1.27 (1.06 – 1.52) |
|  | ≥60 years | 0.99 (0.87 – 1.13) | 1.06 (0.89 – 1.25) | 1.52 (1.18 – 1.95) |
| Gender | Female | 1.00 (Reference) | 1.00 (Reference) | 1.00 (Reference) |
|  | Male | Not entered | 0.92 (0.84 – 0.99) | 0.85 (0.75 – 0.97) |
| Population category | General population | 1.00 (Reference) | Not entered | Not entered |
|  | Students | 1.07 (0.99 – 1.15) | Not entered | Not entered |
|  | High-risk occupations | 1.27 (0.98 – 1.64) | Not entered | Not entered |
|  | Very high-risk occupations | 0.54 (0.33 – 0.88) | Not entered | Not entered |
| Previous rabies vaccination (human) | No | 1.00 (Reference) | 1.00 (Reference) | Not entered |
|  | Yes | 1.17 (1.11 – 1.23) | 0.84 (0.77 – 0.92) | Not entered |
|  | Unknown | 1.11 (0.69 – 1.79) | 1.01 (0.48 – 2.11) | Not entered |
| Injuring animal category | Other animals | 1.00 (Reference) | 1.00 (Reference) | Not estimable |
|  | Cats/Dogs | 0.96 (0.40 – 2.33) | 1.70 (1.33 – 2.16) | Not estimable |
|  | Bats | 0.96 (0.35 – 2.61) | 1.22 (0.40 – 3.70) | Not estimable |
| Animal source | Domestic | 1.00 (Reference) | 1.00 (Reference) | 1.00 (Reference) |
|  | Stray | 0.90 (0.84 – 0.97) | 0.60 (0.41 – 0.87) | 0.53 (0.30 – 0.94) |
|  | Unknown | 0.73 (0.60 – 0.88) | 0.77 (0.69 – 0.87) | 0.77 (0.64 – 0.92) |
|  | Wild | 1.02 (0.75 – 1.38) | 1.61 (0.98 – 2.63) | 1.06 (0.52 – 2.16) |
| Animal vaccination history (animal) | Vaccinated | 1.00 (Reference) | 1.00 (Reference) | 1.00 (Reference) |
|  | Unknown | 0.93 (0.87 – 0.99) | 1.34 (1.20 – 1.49) | 1.23 (1.05 – 1.45) |
|  | Unvaccinated | 0.79 (0.73 – 0.86) | 1.58 (1.40 – 1.79) | 1.46 (1.21 – 1.76) |
| Injury type | Bite/Scratch/Lick on broken skin | 1.00 (Reference) | Not entered | Not estimable |
|  | Contact with intact skin | 1.92 (0.66 – 5.59) | Not entered | Not estimable |
|  | Open wound/mucosa contact | 1.91 (1.52 – 2.41) | Not entered | Not estimable |
|  | Other | 0.99 (0.78 – 1.24) | Not entered | Not estimable |
| Exposure site | Head/Neck | 1.00 (Reference) | Not entered | Not entered |
|  | Trunk/Limbs | 0.92 (0.81 – 1.04) | Not entered | Not entered |
| Exposure grade | Grade II | 1.00 (Reference) | 1.00 (Reference) | 1.00 (Reference) |
|  | Grade III | 0.69 (0.65 – 0.73) | 0.67 (0.62 – 0.73) | 0.62 (0.55 – 0.71) |
| Wound management | Adequate | 1.00 (Reference) | 1.00 (Reference) | 1.00 (Reference) |
|  | Inadequate | 1.57 (1.31 – 1.88) | 1.75 (1.26 – 2.44) | 1.62 (0.98 – 2.68) |
| Weight | Continuous | Not entered | Not entered | Not entered |

Notes: aOR, adjusted odds ratio; CI, confidence interval. "Not entered" indicates the variable was not entered into the multivariable model (*P* ≥ 0.1 in univariate analysis).

"Not estimable" indicates that the variable was excluded from the 48-hour analysis because one or more subgroups had zero events in the delay group. Weight was not entered into the multivariable model at any of the three thresholds. All models used the same variable selection strategy as the primary analysis (univariate *P* < 0.1 for entry into multivariable model).
